# Statistical tests to compare motif count exceptionalities

**DOI:** 10.1186/1471-2105-8-84

**Published:** 2007-03-08

**Authors:** Stéphane Robin, Sophie Schbath, Vincent Vandewalle

**Affiliations:** 1 INA PG/ENGREF/INRA, UMR518 Unité Mathématiques et Informatique Appliquées, 75005 Paris, France; 2 INRA, UR1077 Unité Mathématique, Informatique et Génome, 78350 Jouy-en-Josas, France

## Abstract

**Background:**

Finding over- or under-represented motifs in biological sequences is now a common task in genomics. Thanks to *p*-value calculation for motif counts, exceptional motifs are identified and represent candidate functional motifs. The present work addresses the related question of comparing the exceptionality of one motif in two different sequences. Just comparing the motif count *p*-values in each sequence is indeed not sufficient to decide if this motif is significantly more exceptional in one sequence compared to the other one. A statistical test is required.

**Results:**

We develop and analyze two statistical tests, an exact binomial one and an asymptotic likelihood ratio test, to decide whether the exceptionality of a given motif is equivalent or significantly different in two sequences of interest. For that purpose, motif occurrences are modeled by Poisson processes, with a special care for overlapping motifs. Both tests can take the sequence compositions into account. As an illustration, we compare the octamer exceptionalities in the *Escherichia coli *K-12 backbone versus variable strain-specific loops.

**Conclusion:**

The exact binomial test is particularly adapted for small counts. For large counts, we advise to use the likelihood ratio test which is asymptotic but strongly correlated with the exact binomial test and very simple to use.

## Background

Detecting motifs with a significantly unexpected frequency in DNA sequences has become a very common task in genome analysis. It is generally addressed to propose candidate functional motifs based on their statistical properties [1-3]. Lots of statistical methods have been developed to that purpose (see the recent surveys by [4] or [5] and references therein) and satisfactory solutions exist now to find exceptional motifs thanks to *p*-value calculations.

More recently, a new related question has arisen in the literature concerning the comparison of motif exceptionalities in two sequences. One wants for instance to compare particular sets of genes [6], upstream regions of CDSs versus whole chromosome [7], structural domains [8], CDSs versus intergenic regions, conserved regions versus strain-specific regions of bacterial genomes [9], or chromosomes from the same species [10]. Chromosomes from different species can also be compared from a comparative genomics point of view. In all these works, one would like to know if a given motif is significantly more exceptional in one sequence compared to another one. This criterion is usually used to identify motifs which are specific from some regions or expected to be more frequent in some particular parts of the genome. Transcription factor binding sites, for instance, are expected to be more frequent in upstream regions than along the whole genome.

Surprisingly, no rigorous statistical method has been proposed yet to decide if a given motif, exact or not, is significantly more exceptional in one sequence compared to a second one. Of course, two *p*-values can be calculated separately on each sequence to know if the motif is exceptional in these sequences but the difficult point is how to compare these two *p*-values from a statistical point of view. It is indeed not sufficient to make the difference or the ratio to know if the two *p*-values are significantly different; One needs a statistical test.

In this paper, we propose two statistical tests to compare the motif count exceptionalities in two independent sequences. In the Results Section, we first present the underlying model for motif occurrences and the null hypothesis to test, namely the motif is similarly exceptional in both sequences. Then we derive an exact binomial test and an asymptotic likelihood ratio test adapted for frequent motifs. Usage conditions and power of both tests are described in the Discussion Section, together with a more refined model for occurrences of overlapping words and the associated tests. An illustration of the method is finally given; We compare the octamer exceptionalities in two sets of regions (backbone/loops) from the *Escherichia coli *K12 leading strands. These two sets correspond to the mosaic structure of *E. coli*'s genome when comparing the two strains K12 and O157:H7: the backbone represents the common regions whereas the loops are specific to the K12 strain. As a toy example all along this paper, we will treat in detail the case of the palindromic octamer cagcgctg which occurs respectively 30 times in the loops (758434 bps long) and 113 times in the backbone (3 882 513 bps long).

## Results

### Poisson model

In sequence *i*, the motif count *N*_*i *_is supposed to have a Poisson distribution with mean (and variance) *λ*_*i*_. This distribution has been shown to fit correctly theoretical (in Markovian sequences, for example) as well as observed count distributions of non-overlapping words [11]; A non-overlapping word is a word such that two occurrences of itself can not overlap in a sequence.

The mean count *λ*_*i *_in sequence *i *must account for three parameters: (*i*) the length ℓ_*i *_of the sequence, (*ii*) the composition of the sequence, (*iii*) the possible exceptionality of the motif in the sequence.

#### Expected intensity

The composition of the sequence can be accounted for via the probability *μ*_*i *_for the motif to occur at any position in the sequence under a simple model. The most popular models are Markov chain models which can fit the frequencies in mono-, di-, tri-nucleotides, etc. Indeed, the Markov chain model of order *m *(denoted by M*m*) takes the (*m *+ 1)-mer composition into account. Under such models, the occurrence probability *μ*_*i *_of a *h*-letter motif **w **= *w*_1 _*w*_2 _... *w*_*h *_on the {a, c, g, t} alphabet can be expressed in terms of counts of its subwords of length *m *and *m *+ 1 [5]. For instance, here are the expression of *μ*_*i *_in models M0, M1 and M(*h *- 2) which fit respectively the composition in bases, in dinucleotides and in oligonucleotides of length *h *- 1:

M0:μi=∏j=1hNi(wj)ℓih,M1:μi=∏j=1h−1Ni(wjwj+1)ℓi∏j=2h−1Ni(wj),M(h−2):μi=Ni(w1⋯wh−1)Ni(w2⋯wh)(ℓi−h+3)Ni(w2⋯wh−1),
MathType@MTEF@5@5@+=feaafiart1ev1aaatCvAUfKttLearuWrP9MDH5MBPbIqV92AaeXatLxBI9gBaebbnrfifHhDYfgasaacH8akY=wiFfYdH8Gipec8Eeeu0xXdbba9frFj0=OqFfea0dXdd9vqai=hGuQ8kuc9pgc9s8qqaq=dirpe0xb9q8qiLsFr0=vr0=vr0dc8meaabaqaciaacaGaaeqabaqabeGadaaakeaafaqaaeWacaaabaGaeeyta0KaeGimaaJaeiOoaOdabaacciGae8hVd02aaSbaaSqaaiabdMgaPbqabaGccqGH9aqpdaWcaaqaamaaradabaGaemOta40aaSbaaSqaaiabdMgaPbqabaGccqGGOaakcqWG3bWDdaWgaaWcbaGaemOAaOgabeaakiabcMcaPaWcbaGaemOAaOMaeyypa0JaeGymaedabaGaemiAaGganiabg+GivdaakeaacqWItecBdaqhaaWcbaGaemyAaKgabaGaemiAaGgaaaaakiabcYcaSaqaaiabb2eanjabigdaXiabcQda6aqaaiab=X7aTnaaBaaaleaacqWGPbqAaeqaaOGaeyypa0ZaaSaaaeaadaqeWaqaaiabd6eaonaaBaaaleaacqWGPbqAaeqaaOGaeiikaGIaem4DaC3aaSbaaSqaaiabdQgaQbqabaGccqWG3bWDdaWgaaWcbaGaemOAaOMaey4kaSIaeGymaedabeaakiabcMcaPaWcbaGaemOAaOMaeyypa0JaeGymaedabaGaemiAaGMaeyOeI0IaeGymaedaniabg+GivdaakeaacqWItecBdaWgaaWcbaGaemyAaKgabeaakmaaradabaGaemOta40aaSbaaSqaaiabdMgaPbqabaGccqGGOaakcqWG3bWDdaWgaaWcbaGaemOAaOgabeaakiabcMcaPaWcbaGaemOAaOMaeyypa0JaeGOmaidabaGaemiAaGMaeyOeI0IaeGymaedaniabg+GivdaaaOGaeiilaWcabaGaeeyta0KaeiikaGIaemiAaGMaeyOeI0IaeGOmaiJaeiykaKIaeiOoaOdabaGae8hVd02aaSbaaSqaaiabdMgaPbqabaGccqGH9aqpdaWcaaqaaiabd6eaonaaBaaaleaacqWGPbqAaeqaaOGaeiikaGIaem4DaC3aaSbaaSqaaiabigdaXaqabaGccqWIVlctcqWG3bWDdaWgaaWcbaGaemiAaGMaeyOeI0IaeGymaedabeaakiabcMcaPiabd6eaonaaBaaaleaacqWGPbqAaeqaaOGaeiikaGIaem4DaC3aaSbaaSqaaiabikdaYaqabaGccqWIVlctcqWG3bWDdaWgaaWcbaGaemiAaGgabeaakiabcMcaPaqaaiabcIcaOiabloriSnaaBaaaleaacqWGPbqAaeqaaOGaeyOeI0IaemiAaGMaey4kaSIaeG4mamJaeiykaKIaemOta40aaSbaaSqaaiabdMgaPbqabaGccqGGOaakcqWG3bWDdaWgaaWcbaGaeGOmaidabeaakiabl+UimjabdEha3naaBaaaleaacqWGObaAcqGHsislcqaIXaqmaeqaaOGaeiykaKcaaiabcYcaSaaaaaa@B38B@

where *N*_*i *_(·) denotes the count in sequence *i*.

If one does not want to account for the sequence composition (this case will be referred to as model M00), then *μ*_*i *_simply depends on the motif, hence *μ*_1 _= *μ*_2 _= (1/4)^*h*^.

The choice of the Markov chain model depends on the sequence composition one wants to fit. For instance, model M2 is often used for coding DNA sequences to take the codon bias into account. Higher the model order, better the fit, but usually the model order is bounded either by *h *- 2 or because the sequence is too small (the number of parameters to be estimated increases exponentially with the order).

Table 1 gives the expected counts ℓ_*i *_*μ*_*i *_for the motif cagcgctg in the *E. coli *loops/backbone sequences. Since *N*_1 _= 30 and *N*_2 _= 113, we see that this motif is highly over-represented in both sequences under models M00, M0 and M1. However, under the richest possible model (M6), it is over-represented in sequence 1 (loops) but under-represented in sequence 2 (backbone).

**Table 1:** Expected count for cagcgctg in the loops (1) and in the backbone (2) of *E. coli *leading strands under different models.

Model	M00	M0	M1	M6	Count
ℓ_1 _*μ*_1_	11.6	9.4	13.9	24.8	*n*_1 _= 30
ℓ_2 _*μ*_2_	59.2	66.0	106.2	126.1	*n*_2 _= 113

#### Exceptionality coefficient

When the motif is not exceptional with respect to the considered model, the mean count *λ*_*i *_is simply ℓ_*i *_*μ*_*i*_. For exceptional motifs, *i.e*. motifs with an observed count *N*_*i *_far from its expectation ℓ_*i *_*μ*_*i*_, under a given model, the mean count *λ*_*i *_should reflect this exceptionality.

We therefore introduce an exceptionality coefficient *k*_*i *_which allows *λ*_*i *_to be greater (or smaller) than the expected value:

*λ*_*i *_: = *k*_*i *_ℓ_*i *_*μ*_*i*_.

In the following, parameters ℓ_*i *_and *μ*_*i *_will be supposed to be known *a priori*: they can be considered as two correction terms. The inference will only be made on *k*_*i*_.

### Hypothesis testing

Comparing the (potential) exceptionality of a motif in two sequences is equivalent to test the null hypothesis **H**_0 _= {*k*_1 _= *k*_2_}.

We emphasize that the respective values of *k*_1 _and *k*_2 _can be larger than one (unexpectedly frequent motif), smaller than one (unexpectedly rare motif) or close to one (motif with expected count). These values do not matter: our only concern is to know if they are significantly different or not.

### Exact binomial test

We first propose an exact test based on a general property of the Poisson distribution. If *N*_1 _and *N*_2 _are two independent Poisson counts with respective means *λ*_1 _and *λ*_2_, the distribution of *N*_1 _given their sum *N*_+ _: = *N*_1 _+ *N*_2 _is binomial [12]: *N*_1 _~ ℬ
 MathType@MTEF@5@5@+=feaafiart1ev1aaatCvAUfKttLearuWrP9MDH5MBPbIqV92AaeXatLxBI9gBaebbnrfifHhDYfgasaacH8akY=wiFfYdH8Gipec8Eeeu0xXdbba9frFj0=OqFfea0dXdd9vqai=hGuQ8kuc9pgc9s8qqaq=dirpe0xb9q8qiLsFr0=vr0=vr0dc8meaabaqaciaacaGaaeqabaqabeGadaaakeaat0uy0HwzTfgDPnwy1egaryqtHrhAL1wy0L2yHvdaiqaacqWFSeIqaaa@377D@ (*N*_+_, *π*) with

π=λ1λ1+λ2=(k1/k2)ℓ1μ1(k1/k2)ℓ1μ1+ℓ2μ2.
 MathType@MTEF@5@5@+=feaafiart1ev1aaatCvAUfKttLearuWrP9MDH5MBPbIqV92AaeXatLxBI9gBaebbnrfifHhDYfgasaacH8akY=wiFfYdH8Gipec8Eeeu0xXdbba9frFj0=OqFfea0dXdd9vqai=hGuQ8kuc9pgc9s8qqaq=dirpe0xb9q8qiLsFr0=vr0=vr0dc8meaabaqaciaacaGaaeqabaqabeGadaaakeaaiiGacqWFapaCcqGH9aqpdaWcaaqaaiab=T7aSnaaBaaaleaacqaIXaqmaeqaaaGcbaGae83UdW2aaSbaaSqaaiabigdaXaqabaGccqGHRaWkcqWF7oaBdaWgaaWcbaGaeGOmaidabeaaaaGccqGH9aqpdaWcaaqaaiabcIcaOiabdUgaRnaaBaaaleaacqaIXaqmaeqaaOGaei4la8Iaem4AaS2aaSbaaSqaaiabikdaYaqabaGccqGGPaqkcqWItecBdaWgaaWcbaGaeGymaedabeaakiab=X7aTnaaBaaaleaacqaIXaqmaeqaaaGcbaGaeiikaGIaem4AaS2aaSbaaSqaaiabigdaXaqabaGccqGGVaWlcqWGRbWAdaWgaaWcbaGaeGOmaidabeaakiabcMcaPiabloriSnaaBaaaleaacqaIXaqmaeqaaOGae8hVd02aaSbaaSqaaiabigdaXaqabaGccqGHRaWkcqWItecBdaWgaaWcbaGaeGOmaidabeaakiab=X7aTnaaBaaaleaacqaIYaGmaeqaaaaakiabc6caUaaa@5A9B@

Under **H**_0_, we have *π *= *π*_0 _with

π0=ℓ1μ1ℓ1μ1+ℓ2μ2     (1)
 MathType@MTEF@5@5@+=feaafiart1ev1aaatCvAUfKttLearuWrP9MDH5MBPbIqV92AaeXatLxBI9gBaebbnrfifHhDYfgasaacH8akY=wiFfYdH8Gipec8Eeeu0xXdbba9frFj0=OqFfea0dXdd9vqai=hGuQ8kuc9pgc9s8qqaq=dirpe0xb9q8qiLsFr0=vr0=vr0dc8meaabaqaciaacaGaaeqabaqabeGadaaakeaaiiGacqWFapaCdaWgaaWcbaGaeGimaadabeaakiabg2da9maalaaabaGaeS4eHW2aaSbaaSqaaiabigdaXaqabaGccqWF8oqBdaWgaaWcbaGaeGymaedabeaaaOqaaiabloriSnaaBaaaleaacqaIXaqmaeqaaOGae8hVd02aaSbaaSqaaiabigdaXaqabaGccqGHRaWkcqWItecBdaWgaaWcbaGaeGOmaidabeaakiab=X7aTnaaBaaaleaacqaIYaGmaeqaaaaakiaaxMaacaWLjaWaaeWaaeaacqaIXaqmaiaawIcacaGLPaaaaaa@44D7@

because *k*_1 _= *k*_2_. In absence of correction (M00 model) for the sequence composition (*i.e*. *μ*_1 _= *μ*_2_), we have *π*_0 _= ℓ_1_/(ℓ_1 _+ ℓ_2_). If furthermore the two sequences have the same length, we get *π*_0 _= 1/2.

Moreover, the proportion *π *and then the expectation of *N*_1_, increases as the ratio *k*_1_/*k*_2 _increases. Therefore, the *p*-value for the one-sided alternative **H**_1 _= {*k*_1 _> *k*_2_} is *p*_*B *_= Pr {ℬ
 MathType@MTEF@5@5@+=feaafiart1ev1aaatCvAUfKttLearuWrP9MDH5MBPbIqV92AaeXatLxBI9gBaebbnrfifHhDYfgasaacH8akY=wiFfYdH8Gipec8Eeeu0xXdbba9frFj0=OqFfea0dXdd9vqai=hGuQ8kuc9pgc9s8qqaq=dirpe0xb9q8qiLsFr0=vr0=vr0dc8meaabaqaciaacaGaaeqabaqabeGadaaakeaat0uy0HwzTfgDPnwy1egaryqtHrhAL1wy0L2yHvdaiqaacqWFSeIqaaa@377D@ (*n*_+_, *π*_0_) ≥ *n*_1_}, i.e.

pB=1−∑d=0n1−1(n+d)π0d(1−π0)n+−d
 MathType@MTEF@5@5@+=feaafiart1ev1aaatCvAUfKttLearuWrP9MDH5MBPbIqV92AaeXatLxBI9gBaebbnrfifHhDYfgasaacH8akY=wiFfYdH8Gipec8Eeeu0xXdbba9frFj0=OqFfea0dXdd9vqai=hGuQ8kuc9pgc9s8qqaq=dirpe0xb9q8qiLsFr0=vr0=vr0dc8meaabaqaciaacaGaaeqabaqabeGadaaakeaacqWGWbaCdaWgaaWcbaGaemOqaieabeaakiabg2da9iabigdaXiabgkHiTmaaqahabaWaaeWaaeaafaqabeGabaaabaGaemOBa42aaSbaaSqaaiabgUcaRaqabaaakeaacqWGKbazaaaacaGLOaGaayzkaaaaleaacqWGKbazcqGH9aqpcqaIWaamaeaacqWGUbGBdaWgaaadbaGaeGymaedabeaaliabgkHiTiabigdaXaqdcqGHris5aGGacOGae8hWda3aa0baaSqaaiabicdaWaqaaiabdsgaKbaakiabcIcaOiabigdaXiabgkHiTiab=b8aWnaaBaaaleaacqaIWaamaeqaaOGaeiykaKYaaWbaaSqabeaacqWGUbGBdaWgaaadbaGaey4kaScabeaaliabgkHiTiabdsgaKbaaaaa@5129@

where *n*_+ _and *n*_1 _are the observed values of *N*_+ _and *N*_1_.

Table 2 gives the probability *π*_0 _and the *p*-value *p*_*B *_for the motif cagcgctg in *E. coli*. At level 5%, the null hypothesis is accepted under models M00 and M6 meaning that the motif is similarly exceptional in both sequences with respect to their length and/or 7-mer composition. However, {*k*_1 _= *k*_2_} is rejected at level 5% against {*k*_1 _> *k*_2_} under models M0 and M1; since cagcgctg is over-represented in both sequences, it means that it is significantly more exceptionally over-represented in sequence 1 (loops) with respect to the base and/or dinucleotide compositions of both sequences.

**Table 2:** Probability *π*_0 _and *p*-value *p*_*B *_under different models for cagcgctg in the *E. coli *loops/backbone comparison.

Model	M00	M0	M1	M6
*π*_0 _(%)	16.3	12.4	11.6	16.4
*p*_*B*_	8.6 10^-2^	2.7 10^-3^	9.1 10^-4^	9.1 10^-2^

### Likelihood ratio test (LRT)

Another test statistic based on the comparison of the likelihood of the data under the **H**_0 _and the alternative hypothesis **H**_1 _= {*k*_1 _≠ *k*_2_} can be derived. This statistic is known as the Likelihood Ratio Test (see [13], vol. IV). In our model (see the Methods Section), it is defined as

LRT=2[N1ln⁡(N1/N+π0)+N2ln⁡(N2/N+1−π0)]
 MathType@MTEF@5@5@+=feaafiart1ev1aaatCvAUfKttLearuWrP9MDH5MBPbIqV92AaeXatLxBI9gBaebbnrfifHhDYfgasaacH8akY=wiFfYdH8Gipec8Eeeu0xXdbba9frFj0=OqFfea0dXdd9vqai=hGuQ8kuc9pgc9s8qqaq=dirpe0xb9q8qiLsFr0=vr0=vr0dc8meaabaqaciaacaGaaeqabaqabeGadaaakeaacqWGmbatcqWGsbGucqWGubavcqGH9aqpcqaIYaGmdaWadaqaaiabd6eaonaaBaaaleaacqaIXaqmaeqaaOGagiiBaWMaeiOBa42aaeWaaeaadaWcaaqaaiabd6eaonaaBaaaleaacqaIXaqmaeqaaOGaei4la8IaemOta40aaSbaaSqaaiabgUcaRaqabaaakeaaiiGacqWFapaCdaWgaaWcbaGaeGimaadabeaaaaaakiaawIcacaGLPaaacqGHRaWkcqWGobGtdaWgaaWcbaGaeGOmaidabeaakiGbcYgaSjabc6gaUnaabmaabaWaaSaaaeaacqWGobGtdaWgaaWcbaGaeGOmaidabeaakiabc+caViabd6eaonaaBaaaleaacqGHRaWkaeqaaaGcbaGaeGymaeJaeyOeI0Iae8hWda3aaSbaaSqaaiabicdaWaqabaaaaaGccaGLOaGaayzkaaaacaGLBbGaayzxaaaaaa@54CC@

where *π*_0 _is defined in (1). Under the null hypothesis, its asymptotic distribution is a chi-square distribution with one degree of freedom.

This test is two-sided, because, under **H**_**1**_, parameters *k*_1 _and *k*_2 _are estimated independently (in particular, without the constraint *k*_1 _> *k*_2_). The exact distribution of *LRT *could be calculated via permutation techniques but the computation time would be tremendeous for large counts. We will then calculate the following asymptotic *p*-value:

pL=Pr⁡{χ2≥2[n1ln⁡(n1/n+π0)+n2ln⁡(n2/n+1−π0)]},
 MathType@MTEF@5@5@+=feaafiart1ev1aaatCvAUfKttLearuWrP9MDH5MBPbIqV92AaeXatLxBI9gBaebbnrfifHhDYfgasaacH8akY=wiFfYdH8Gipec8Eeeu0xXdbba9frFj0=OqFfea0dXdd9vqai=hGuQ8kuc9pgc9s8qqaq=dirpe0xb9q8qiLsFr0=vr0=vr0dc8meaabaqaciaacaGaaeqabaqabeGadaaakeaacqWGWbaCdaWgaaWcbaGaemitaWeabeaakiabg2da9iGbccfaqjabckhaYnaacmqabaacciGae83Xdm2aaWbaaSqabeaacqaIYaGmaaGccqGHLjYScqaIYaGmdaWadaqaaiabd6gaUnaaBaaaleaacqaIXaqmaeqaaOGagiiBaWMaeiOBa42aaeWaaeaadaWcaaqaaiabd6gaUnaaBaaaleaacqaIXaqmaeqaaOGaei4la8IaemOBa42aaSbaaSqaaiabgUcaRaqabaaakeaacqWFapaCdaWgaaWcbaGaeGimaadabeaaaaaakiaawIcacaGLPaaacqGHRaWkcqWGUbGBdaWgaaWcbaGaeGOmaidabeaakiGbcYgaSjabc6gaUnaabmaabaWaaSaaaeaacqWGUbGBdaWgaaWcbaGaeGOmaidabeaakiabc+caViabd6gaUnaaBaaaleaacqGHRaWkaeqaaaGcbaGaeGymaeJaeyOeI0Iae8hWda3aaSbaaSqaaiabicdaWaqabaaaaaGccaGLOaGaayzkaaaacaGLBbGaayzxaaaacaGL7bGaayzFaaGaeiilaWcaaa@5FD6@

where *n*_2 _is the observed value of *N*_2 _and *χ*^2 ^~ *χ*^2 ^(1).

Table 3 gives the LRT statistic and the associated *p*-value for the motif cagcgctg in *E. coli*. Remember that the *LRT *is two-sided, so *p*_*L *_have to be divided by two when compared to the one-sided binomial *p*-value *p*_*B*_. We see that the significances obtained with the *LRT *are different from the ones obtained with the exact binomial test, but the qualitative conclusions are the same.

**Table 3:** *LRT *statistic and associated *p*-value *p*_*L *_under different models for cagcgctg in the *E. coli *loops/backbone comparison.

Model	M00	M0	M1	M6
*LRT*	2.1	8.2	10.2	2.0
*p*_*L*_	1.5 10^-1^	4.2 10^-3^	1.4 10^-3^	1.6 10^-1^

#### Chi-square test

Another standard asymptotic test is the chi-square test where the counts *N*_*i *_are compared to their expected values N^
 MathType@MTEF@5@5@+=feaafiart1ev1aaatCvAUfKttLearuWrP9MDH5MBPbIqV92AaeXatLxBI9gBaebbnrfifHhDYfgasaacH8akY=wiFfYdH8Gipec8Eeeu0xXdbba9frFj0=OqFfea0dXdd9vqai=hGuQ8kuc9pgc9s8qqaq=dirpe0xb9q8qiLsFr0=vr0=vr0dc8meaabaqaciaacaGaaeqabaqabeGadaaakeaadaqiaaqaaiabd6eaobGaayPadaaaaa@2E93@_*i *_under **H**_0 _given the total count *N*_+_:

X2=∑i=12(Ni−N^i)2N^i
 MathType@MTEF@5@5@+=feaafiart1ev1aaatCvAUfKttLearuWrP9MDH5MBPbIqV92AaeXatLxBI9gBaebbnrfifHhDYfgasaacH8akY=wiFfYdH8Gipec8Eeeu0xXdbba9frFj0=OqFfea0dXdd9vqai=hGuQ8kuc9pgc9s8qqaq=dirpe0xb9q8qiLsFr0=vr0=vr0dc8meaabaqaciaacaGaaeqabaqabeGadaaakeaacqWGybawdaahaaWcbeqaaiabikdaYaaakiabg2da9maaqahabaWaaSaaaeaacqGGOaakcqWGobGtdaWgaaWcbaGaemyAaKgabeaakiabgkHiTmaaHaaabaGaemOta4eacaGLcmaadaWgaaWcbaGaemyAaKgabeaakiabcMcaPmaaCaaaleqabaGaeGOmaidaaaGcbaWaaecaaeaacqWGobGtaiaawkWaamaaBaaaleaacqWGPbqAaeqaaaaaaeaacqWGPbqAcqGH9aqpcqaIXaqmaeaacqaIYaGma0GaeyyeIuoaaaa@4402@

where N^
 MathType@MTEF@5@5@+=feaafiart1ev1aaatCvAUfKttLearuWrP9MDH5MBPbIqV92AaeXatLxBI9gBaebbnrfifHhDYfgasaacH8akY=wiFfYdH8Gipec8Eeeu0xXdbba9frFj0=OqFfea0dXdd9vqai=hGuQ8kuc9pgc9s8qqaq=dirpe0xb9q8qiLsFr0=vr0=vr0dc8meaabaqaciaacaGaaeqabaqabeGadaaakeaadaqiaaqaaiabd6eaobGaayPadaaaaa@2E93@_1 _= *π*_0 _*N*_+ _and N^
 MathType@MTEF@5@5@+=feaafiart1ev1aaatCvAUfKttLearuWrP9MDH5MBPbIqV92AaeXatLxBI9gBaebbnrfifHhDYfgasaacH8akY=wiFfYdH8Gipec8Eeeu0xXdbba9frFj0=OqFfea0dXdd9vqai=hGuQ8kuc9pgc9s8qqaq=dirpe0xb9q8qiLsFr0=vr0=vr0dc8meaabaqaciaacaGaaeqabaqabeGadaaakeaadaqiaaqaaiabd6eaobGaayPadaaaaa@2E93@_2 _= (1 - *π*_0_)*N*_+_. Under the null hypothesis, *X*^2 ^has also an asymptotic chi-square distribution with one degree of freedom. It is also an intrinsically two-sided test. Further analyzes (including simulations) not presented here (see [14]) show that this test performs very similarly to the LRT in every situations. Note that the chi-square test is the same as the score test [13].

## Discussion

### LRT distribution

The chi-square distribution of the LRT statistic is only asymptotic, so the actual level may be different from the nominal one (typically *α *= 5%). To measure this difference, we have calculated this actual level for different values of *π*_0 _and *N*_+_. Since *LRT *is a function of *N*_1_, the actual level can be derived from the exact distribution of *N*_1 _given *N*_+ _which is binomial (see Results Section).

**Figure 1:**
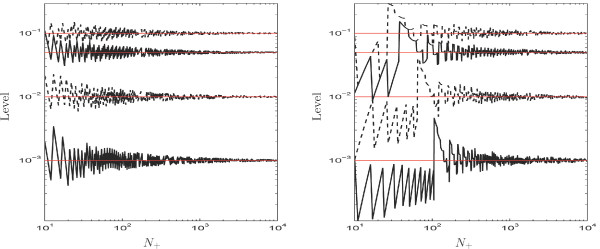
Actual level (log scale) of the LRT as a function of *N*_+ _(log scale) for a nominal level *α *= 0.1%, 1%, 5% or 10% and probability *π*_0 _= 0.5 (left) and 0.95 (right). (Since the LRT test is two-sided, the right plot also holds for *π*_0 _= 0.05).

Figure 1 compares both levels (actual and nominal). Since the counts are discrete, the actual level can never be exactly *α *leading to oscillations in the plot. We see that the nominal level is only reached with *N*_+ _≃ 1000 for *π*_0 _= 0.5 and even later for *π*_0 _= 0.95 (or *π*_0 _= 0.05). It means that the chi-square approximation of the LRT statistics is only valid for motifs with many total occurrences.

Regarding the motif cagcgctg, because *π*_0 _is about 15% (cf. Table 2), the picture is close to the right plot of Figure 1; In fact, with a total count of 143, the actual level is respectively 0.095%, 1.1%, 5.1% and 12.5% for a nominal level *α *equal to 0.1%, 1%, 5% and 10%.

#### LRT as a contrast measure

The LRT statistic can still be used as a contrast measure, i.e. a measure of the difference, between the two exceptionalities. For large values of *N*_+ _it is much faster and easier to compute than the binomial *p*-value. We will see in the illustration below that the two quantities are strongly correlated.

### Decidability limits for the binomial test

Because the binomial test is exact, the actual and nominal levels are equal. The significance can then always be determined. It would be maximal when *N*_1 _= *N*_+ _(*i.e*. *N*_2 _= 0) and the corresponding *p*-value *p*_*B *_would be equal to π0N+
 MathType@MTEF@5@5@+=feaafiart1ev1aaatCvAUfKttLearuWrP9MDH5MBPbIqV92AaeXatLxBI9gBaebbnrfifHhDYfgasaacH8akY=wiFfYdH8Gipec8Eeeu0xXdbba9frFj0=OqFfea0dXdd9vqai=hGuQ8kuc9pgc9s8qqaq=dirpe0xb9q8qiLsFr0=vr0=vr0dc8meaabaqaciaacaGaaeqabaqabeGadaaakeaaiiGacqWFapaCdaqhaaWcbaGaeGimaadabaGaemOta40aaSbaaWqaaiabgUcaRaqabaaaaaaa@31BF@. Therefore, if this minimal *p*-value is greater than the desired level *α *(typically 5%), no significance conclusion can be made. This happens when π0N+
 MathType@MTEF@5@5@+=feaafiart1ev1aaatCvAUfKttLearuWrP9MDH5MBPbIqV92AaeXatLxBI9gBaebbnrfifHhDYfgasaacH8akY=wiFfYdH8Gipec8Eeeu0xXdbba9frFj0=OqFfea0dXdd9vqai=hGuQ8kuc9pgc9s8qqaq=dirpe0xb9q8qiLsFr0=vr0=vr0dc8meaabaqaciaacaGaaeqabaqabeGadaaakeaaiiGacqWFapaCdaqhaaWcbaGaeGimaadabaGaemOta40aaSbaaWqaaiabgUcaRaqabaaaaaaa@31BF@*α*, *i.e*. when *N*_+ _≥ ln (*α*)/ln(*π*_0_).

**Figure 2:**
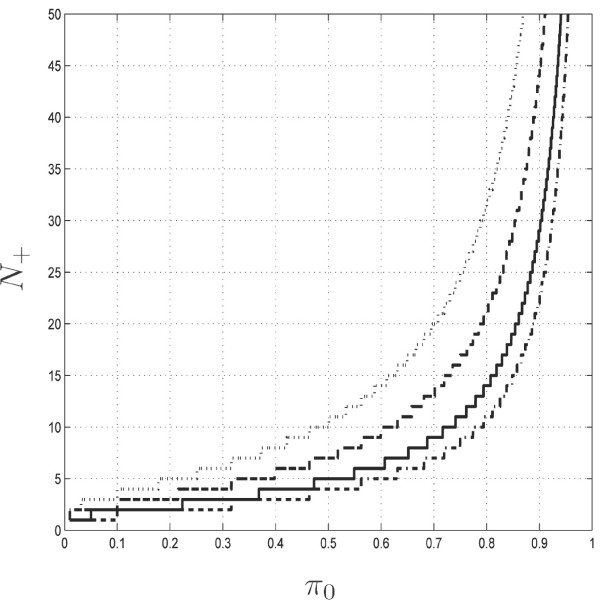
Minimal count *N*_+ _to get a significant result with the binomial test as a function of the probability *π*_0_. Curves correspond to different levels *α *= 0.1%, 1%, 5% or 10% (from top to bottom).

Figure 2 gives this critical value of *N*_+ _for various values of *π*_0 _and *α*. We see, for instance, that for *π*_0 _= 0.7 and *N*_+ _= 10, one may get significant results at a level greater than 5% but not at a level smaller than 1%.

### Power

An important property for a statistical test is its ability to detect departure from the null hypothesis. This ability is measured by the power of the test which is the probability to exceed the significance threshold (defined under **H**_0_) when the true parameter satisfies **H**_1_. In our case, the parameter of interest is

π=λ1λ1+λ2=(k1/k2)ℓ1μ1(k1/k2)ℓ1μ1+ℓ2μ2
 MathType@MTEF@5@5@+=feaafiart1ev1aaatCvAUfKttLearuWrP9MDH5MBPbIqV92AaeXatLxBI9gBaebbnrfifHhDYfgasaacH8akY=wiFfYdH8Gipec8Eeeu0xXdbba9frFj0=OqFfea0dXdd9vqai=hGuQ8kuc9pgc9s8qqaq=dirpe0xb9q8qiLsFr0=vr0=vr0dc8meaabaqaciaacaGaaeqabaqabeGadaaakeaaiiGacqWFapaCcqGH9aqpdaWcaaqaaiab=T7aSnaaBaaaleaacqaIXaqmaeqaaaGcbaGae83UdW2aaSbaaSqaaiabigdaXaqabaGccqGHRaWkcqWF7oaBdaWgaaWcbaGaeGOmaidabeaaaaGccqGH9aqpdaWcaaqaaiabcIcaOiabdUgaRnaaBaaaleaacqaIXaqmaeqaaOGaei4la8Iaem4AaS2aaSbaaSqaaiabikdaYaqabaGccqGGPaqkcqWItecBdaWgaaWcbaGaeGymaedabeaakiab=X7aTnaaBaaaleaacqaIXaqmaeqaaaGcbaGaeiikaGIaem4AaS2aaSbaaSqaaiabigdaXaqabaGccqGGVaWlcqWGRbWAdaWgaaWcbaGaeGOmaidabeaakiabcMcaPiabloriSnaaBaaaleaacqaIXaqmaeqaaOGae8hVd02aaSbaaSqaaiabigdaXaqabaGccqGHRaWkcqWItecBdaWgaaWcbaGaeGOmaidabeaakiab=X7aTnaaBaaaleaacqaIYaGmaeqaaaaaaaa@59AD@

which is equal to *π*_0 _when *k*_1 _= *k*_2_. So the departure from **H**_0 _will be measured by the ratio *k*_1_/*k*_2 _when it differs from 1.

#### Exact binomial

**Figure 3:**
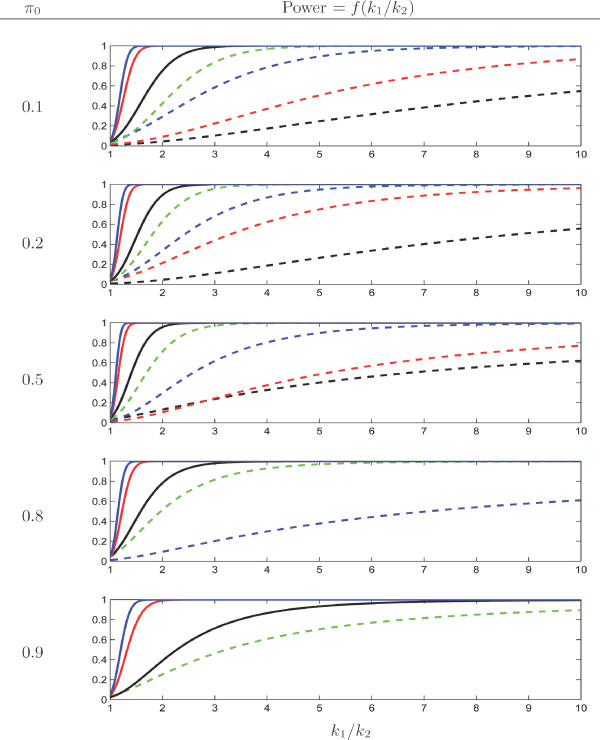
Power of the exact binomial test with level *α *= 5% as a function of *k*_1_/*k*_2 _(*x*-axis) for different values of *π*_0_. Curves correspond to different values of the total count *N*_+ _= 5 (dashed black), 10 (dashed red), 20 (dashed blue), 50 (dashed green), 100 (solid black), 500 (solid red) and 1000 (solid blue). Missing curves correspond to the values of *N*_+ _for which no significant results at level *α *= 5% can be obtained (cf. the Discussion Section).

Figure 3 presents the power of the exact binomial test when *k*_1_/*k*_2 _increases. As expected, the power increases with *N*_+_. Moreover, it decreases when *π*_0 _increases i.e. when the expected ratio ℓ_1 _*μ*_1_/(ℓ_2 _*μ*_2_) increases. It means that, when the motif is already expected to be much more frequent in sequence 1 than in sequence 2, it is more difficult to detect that its exceptionality in the first sequence is also higher.

The motif cagcgctg occurs *N*_+ _= 143 times in the whole genome. In the different models considered in Table 2, probability *π*_0 _is between 11.6% and 16.4%. The power of the binomial test in this case can therefore be read in Figure 3, in the two top plots between the black and red solid lines. We see that a ratio *k*_1_/*k*_2 _= 2 can be detected with probability greater than 90%, while a ratio of 1.5 will be detected with a bit more than 50% probability.

#### LRT

The same analysis can be made for the LRT tests. However, this only makes sense for sufficiently large *N*_+_, to guaranty the validity of the chi-square distribution.

### Case of overlapping words

#### Compound Poisson model

The distribution of overlapping word occurrences is better modeled by a compound Poisson process (see [15]) in the following way:

• The word occurs in clumps distributed according to a Poisson process. The number of clumps *C*_*i *_in sequence *i *is hence a random Poisson variable with mean denoted by λ˜
 MathType@MTEF@5@5@+=feaafiart1ev1aaatCvAUfKttLearuWrP9MDH5MBPbIqV92AaeXatLxBI9gBaebbnrfifHhDYfgasaacH8akY=wiFfYdH8Gipec8Eeeu0xXdbba9frFj0=OqFfea0dXdd9vqai=hGuQ8kuc9pgc9s8qqaq=dirpe0xb9q8qiLsFr0=vr0=vr0dc8meaabaqaciaacaGaaeqabaqabeGadaaakeaaiiGacuWF7oaBgaacaaaa@2E76@_*i*_.

• The size *V*_*ic *_of the *c*-th clump (in sequence *i*) is random with geometric distribution:

Pr{*V*_*ic *_= *v*} = aiv−1
 MathType@MTEF@5@5@+=feaafiart1ev1aaatCvAUfKttLearuWrP9MDH5MBPbIqV92AaeXatLxBI9gBaebbnrfifHhDYfgasaacH8akY=wiFfYdH8Gipec8Eeeu0xXdbba9frFj0=OqFfea0dXdd9vqai=hGuQ8kuc9pgc9s8qqaq=dirpe0xb9q8qiLsFr0=vr0=vr0dc8meaabaqaciaacaGaaeqabaqabeGadaaakeaacqWGHbqydaqhaaWcbaGaemyAaKgabaGaemODayNaeyOeI0IaeGymaedaaaaa@32D1@ (1 - *a*_*i*_).

The clump sizes are supposed to be independent. Parameter *a*_*i *_is the *overlapping probability *of the motif and can be calculated under various Markovian models (see [5]).

In this setting, the count *N*_*i *_is hence the sum of the sizes of *C*_*i *_clumps and has the Polya-Aeppli (or *geometric Poisson*) distribution (see [12]). We have (see [5]) λ˜
 MathType@MTEF@5@5@+=feaafiart1ev1aaatCvAUfKttLearuWrP9MDH5MBPbIqV92AaeXatLxBI9gBaebbnrfifHhDYfgasaacH8akY=wiFfYdH8Gipec8Eeeu0xXdbba9frFj0=OqFfea0dXdd9vqai=hGuQ8kuc9pgc9s8qqaq=dirpe0xb9q8qiLsFr0=vr0=vr0dc8meaabaqaciaacaGaaeqabaqabeGadaaakeaaiiGacuWF7oaBgaacaaaa@2E76@_*i *_= (1 - *a*_*i*_) *λ*_*i*_. In the case of a non-overlapping word, we have *C*_*i *_= *N*_*i*_, *a*_*i *_= 0 and *λ*_*i *_= *λ*_*i*_. For overlapping words, the mean clump size is equal to 1/(1 - *a*_*i*_) and increases with *a*_*i*_.

#### Tests

An overlapping word can occur with an exceptional frequency (*i*) because of an exceptional number of clumps or (*ii*) because of exceptional sizes of clumps. Then comparing the exceptionalities of an overlapping word in two sequences leads to compare the number of clumps *C*_1 _with *C*_2_, and/or the sizes *V*_1*c*_'s with *V*_2*c*_'s.

##### Comparison of the number of clumps

In this compound Poisson model, the number of clumps in each sequence is Poisson distributed. The comparison of the counts *C*_1 _and *C*_2 _is then exactly equivalent to the comparison of the counts *N*_1 _and *N*_2 _studied in the Results Section, replacing *λ*_*i *_by λ˜
 MathType@MTEF@5@5@+=feaafiart1ev1aaatCvAUfKttLearuWrP9MDH5MBPbIqV92AaeXatLxBI9gBaebbnrfifHhDYfgasaacH8akY=wiFfYdH8Gipec8Eeeu0xXdbba9frFj0=OqFfea0dXdd9vqai=hGuQ8kuc9pgc9s8qqaq=dirpe0xb9q8qiLsFr0=vr0=vr0dc8meaabaqaciaacaGaaeqabaqabeGadaaakeaaiiGacuWF7oaBgaacaaaa@2E76@_*i *_and *μ*_*i *_by μ˜
 MathType@MTEF@5@5@+=feaafiart1ev1aaatCvAUfKttLearuWrP9MDH5MBPbIqV92AaeXatLxBI9gBaebbnrfifHhDYfgasaacH8akY=wiFfYdH8Gipec8Eeeu0xXdbba9frFj0=OqFfea0dXdd9vqai=hGuQ8kuc9pgc9s8qqaq=dirpe0xb9q8qiLsFr0=vr0=vr0dc8meaabaqaciaacaGaaeqabaqabeGadaaakeaaiiGacuWF8oqBgaacaaaa@2E78@_*i *_:= (1 - *a*_*i*_) *μ*_*i*_.

##### Exact test for the overlapping probability under M00

The question is now to test the null hypothesis **H**_0 _= {*a*_1 _= *a*_2_}. This comparison is made conditionally to the observed counts *N*_1 _and *N*_2_. It only makes sense if the motif occurs at least once in each sequence, *i.e*. if *N*_1_, *N*_2_, *C*_1 _and *C*_2 _are all larger than (or equal to) 1. In this case, the first occurrence necessarily corresponds to the first clump and the *C*_*i *_- 1 last clumps have to be chosen among the other *N*_*i *_- 1 motif occurrences. Since a motif occurrence (except the first one) corresponds to a clump occurrence with probability 1 - *a*_*i*_, the number of clumps (except the first one) has a binomial distribution:

*C*_*i *_- 1 ~ ℬ
 MathType@MTEF@5@5@+=feaafiart1ev1aaatCvAUfKttLearuWrP9MDH5MBPbIqV92AaeXatLxBI9gBaebbnrfifHhDYfgasaacH8akY=wiFfYdH8Gipec8Eeeu0xXdbba9frFj0=OqFfea0dXdd9vqai=hGuQ8kuc9pgc9s8qqaq=dirpe0xb9q8qiLsFr0=vr0=vr0dc8meaabaqaciaacaGaaeqabaqabeGadaaakeaat0uy0HwzTfgDPnwy1egaryqtHrhAL1wy0L2yHvdaiqaacqWFSeIqaaa@377D@ (*N*_*i *_- 1, 1 - *a*_*i*_)     (2)

which means that the expected number of clumps decreases when the overlapping probability increases.

Following the same strategy as for the non-overlapping case, we base our test on the distribution of *C*_1 _given the total clump count *C*_+ _= *C*_1 _+ *C*_2_. Under **H**_0_, (*C*_1 _- 1) has an hyper-geometric distribution ℋ
 MathType@MTEF@5@5@+=feaafiart1ev1aaatCvAUfKttLearuWrP9MDH5MBPbIqV92AaeXatLxBI9gBaebbnrfifHhDYfgasaacH8akY=wiFfYdH8Gipec8Eeeu0xXdbba9frFj0=OqFfea0dXdd9vqai=hGuQ8kuc9pgc9s8qqaq=dirpe0xb9q8qiLsFr0=vr0=vr0dc8meaabaqaciaacaGaaeqabaqabeGadaaakeaat0uy0HwzTfgDPnwy1egaryqtHrhAL1wy0L2yHvdaiqaacqWFlecsaaa@3762@ (*N*_+ _- 2, *N*_1 _- 1, *C*_+ _- 2) (see [12], Eq. (3.23)):

Pr⁡{C1=c1|N1,N2,C+}=(N1−1c1−1)(N2−1C+−c1−1)(N+−2C+−2)
 MathType@MTEF@5@5@+=feaafiart1ev1aaatCvAUfKttLearuWrP9MDH5MBPbIqV92AaeXatLxBI9gBaebbnrfifHhDYfgasaacH8akY=wiFfYdH8Gipec8Eeeu0xXdbba9frFj0=OqFfea0dXdd9vqai=hGuQ8kuc9pgc9s8qqaq=dirpe0xb9q8qiLsFr0=vr0=vr0dc8meaabaqaciaacaGaaeqabaqabeGadaaakeaafaqabeGabaaabaGagiiuaaLaeiOCaiNaei4EaSNaem4qam0aaSbaaSqaaiabigdaXaqabaGccqGH9aqpcqWGJbWydaWgaaWcbaGaeGymaedabeaakiabcYha8jabd6eaonaaBaaaleaacqaIXaqmaeqaaOGaeiilaWIaemOta40aaSbaaSqaaiabikdaYaqabaGccqGGSaalcqWGdbWqdaWgaaWcbaGaey4kaScabeaakiabc2ha9jabg2da9aqaamaalaaabaWaaeWaaeaafaqabeGabaaabaGaemOta40aaSbaaSqaaiabigdaXaqabaGccqGHsislcqaIXaqmaeaacqWGJbWydaWgaaWcbaGaeGymaedabeaakiabgkHiTiabigdaXaaaaiaawIcacaGLPaaadaqadaqaauaabeqaceaaaeaacqWGobGtdaWgaaWcbaGaeGOmaidabeaakiabgkHiTiabigdaXaqaaiabdoeadnaaBaaaleaacqGHRaWkaeqaaOGaeyOeI0Iaem4yam2aaSbaaSqaaiabigdaXaqabaGccqGHsislcqaIXaqmaaaacaGLOaGaayzkaaaabaWaaeWaaeaafaqabeGabaaabaGaemOta40aaSbaaSqaaiabgUcaRaqabaGccqGHsislcqaIYaGmaeaacqWGdbWqdaWgaaWcbaGaey4kaScabeaakiabgkHiTiabikdaYaaaaiaawIcacaGLPaaaaaaaaaaa@6402@

The overlapping probability *a*_1 _is then significantly greater than *a*_2 _if the probability Pr{*C*_1 _≤ *c*_1_|*N*_1_, *N*_2_, *C*_+_} is smaller than a given level *α*.

##### Exact test in the general case

The previous test does not account for the composition of the sequences. The overlapping probabilities *a*_1 _and *a*_2 _can be expected to be different, according to some null model. In this case, the true overlapping probability in sequence *i *is *b*_*i *_= *h*_*i *_*a*_*i*_, where *h*_*i *_is an exceptionality coefficient (analogous to *k*_*i *_for the mean count). The problem is then to test **H**_0 _= {*h*_1 _= *h*_2_}. Such a test is proposed in Appendix: it involves the generalized negative hyper-geometric distribution.

##### Asymptotic tests

As for the counts *N *and *C*, asymptotic tests such as likelihood ratio, chi-square or score tests can be derived to compare exceptionalities in terms of overlaps. These tests are not presented here to avoid further statistical developments but also because the small overlapping probabilities generally observed make them rarely relevant.

### Illustration

#### Materials

Comparing complete genomes of strains of single bacterial species allows to determine highly conserved regions (so-called *backbone*) and numerous strain-specific DNA segments (so-called *loops*) for each strain. These mosaic structures help to understand the evolution of bacterial genomes. Indeed, the backbone probably corresponds to the common ancestral strain and is under vertical pressure whereas the loops may be associated with mobile elements or strain-specific pathogenicity. Such backbone/loops segmentation has been systematically performed [9] and store in the public MOSAIC database [16]. We have extracted from this database the *E. coli *K-12 specific loops (sequence 1) and the backbone (sequence 2) obtained from the pairwise alignment of the complete genomes of *E. coli *K-12 laboratory strain and the enterohemorrhagic *E. coli *O157:H7 strain. As an illustration, we have compared the exceptionalities of all the 65536 octamers in the backbone versus in the loops. Such comparison will point out octamers which do not have the same constraint, with respect to their frequency, on the loops versus on the backbone.

#### Exact binomial test

**Figure 4:**
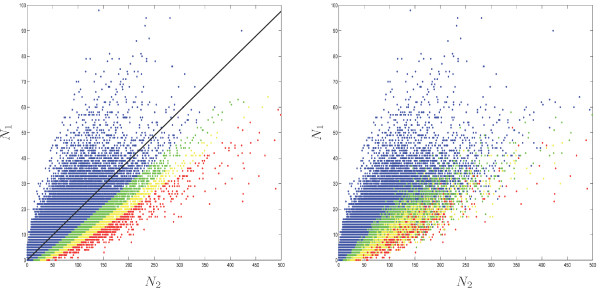
Counts *N*_2 _(*x*-axis: backbone) and *N*_1 _(*y*-axis: loops) for all the octamers under the M00 (left) and M1 (right) models. The color indicates the significance of the binomial test in the M00 model: blue = '*p*_*B *_> 0.01', green = '*p*_*B *_< 0.01', yellow = '*p*_*B *_< 0.001', red = '*p*_*B *_< 0.0001'. The solid black line on the left indicates the expected ratio *N*_1_/*N*_2 _= *π*_0_/(1 - *π*_0_).

Figure 4 presents the significance of the binomial test for all octamers in the backbone/loops comparison. The limits between the different significance levels are clear under M00 because the probability *π*_0 _is the same for all octamers, while they are fuzzy under M1 because *π*_0 _depends on the octamer composition. In this case, same counts (*N*_1_, *N*_2_) may result in different *p*_*B *_values. The distribution of the *p*-value *p*_*B *_is summarized in Table 4. The 10 motifs with smallest *p*-values, i.e. with an exceptionality coefficient significantly higher in the loops than in the backbone, are listed in the top of Table 5. Multiple testing problems arise when we compare the exceptionalities of the 65 536 octamers simultaneously. Table 6 gives the number of significant octamers and the corresponding threshold when adjusting for a False Discovery Rate (FDR, [17]) of 1%. For example, under model M1 only 154 octamers are significantly more exceptional in the loops. These octamers have all a *p*-value *p*_*B *_smaller than 2.2 10^-5^.

**Table 4:** Number of significantly unbalanced octamers under different models and for different thresholds.

		Model:	M00	M0	M1	M6
	*p*_*B*_	< 10^-4^	277	126	83	37
10^-4 ^≤	*p*_*B*_	< 10^-3^	519	303	247	4
10^-3 ^≤	*p*_*B*_	< 10^-2^	1758	1330	1143	104
10^-2 ^≤	*p*_*B*_		62982	63777	64063	65391

**Table 5:** Top: 10 motifs with smallest *p*-value *p*_*B *_(*k*_loops _> *k*_backbone_) for model M00, M0, M1 and M6. * indicates overlapping words. Bottom: 10 motifs with smallest *p*-value p′B
 MathType@MTEF@5@5@+=feaafiart1ev1aaatCvAUfKttLearuWrP9MDH5MBPbIqV92AaeXatLxBI9gBaebbnrfifHhDYfgasaacH8akY=wiFfYdH8Gipec8Eeeu0xXdbba9frFj0=OqFfea0dXdd9vqai=hGuQ8kuc9pgc9s8qqaq=dirpe0xb9q8qiLsFr0=vr0=vr0dc8meaabaqaciaacaGaaeqabaqabeGadaaakeaacuWGWbaCgaqbamaaBaaaleaacqWGcbGqaeqaaaaa@2F5A@ (*k*_backbone _> *k*_loops_).

M00		M0		M1		M6	
cggataag	1.2 10-^19^	cggataag	3.9 10^-20^	cggataag	2.7 10^-18^	gggataaa	2.4 10^-4^
ggataagg*	8.6 10^-16^	ccgcatcc*	2.0 10^-16^	taaggcgt*	9.1 10^-15^	tcgaccaa	3.0 10^-4^
taaggcgt*	4.6 10^-15^	ggataagg*	3.0 10^-16^	ccgcatcc*	4.0 10^-14^	agttttta*	4.5 10^-4^
gataaggc	1.2 10^-14^	tgtaggcc	1.1 10^-15^	acgccgca*	4.0 10^-14^	aagtgata*	5.3 10^-4^
taataaaa	1.9 10^-14^	tcaggcct*	2.9 10^-15^	ataaggcg	3.2 10^-13^	gatagcgc	8.1 10^-4^
ataaggcg	5.6 10^-14^	taaggcgt*	2.9 10^-15^	gccgcatc	1.0 10^-12^	gggtcagg*	1.5 10^-3^
ctgataag	1.2 10^-13^	gataaggc	4.9 10^-15^	gataaggc	2.2 10^-12^	agccgaga*	1.7 10-^3^
tgtaggcc	4.0 10^-13^	ggcctaca	1.1 10^-14^	gttccccg*	4.0 10^-12^	gaggttac	1.7 10^-3^
cttatccg	5.5 10^-13^	ccggccta	1.2 10^-14^	cgcatccg*	4.4 10^-12^	cagagtcc*	1.8 10^-3^
ccttatcc*	6.0 10^-13^	aggcctac	1.4 10^-14^	tgtaggcc	4.7 10^-12^	ccctggcc*	2.0 10^-3^

ggcgctgg*	< 10-^20^	ctggaaga	6.8 10^-10^	ctggaaga	1.2 10^-10^	tcggttac	4.9 10^-4^
gcgctgga	2.5 10^-14^	cgatgaag	2.9 10^-9^	atctggtg	3.3 10^-8^	ggttgatg*	5.4 10^-4^
cggcgctg	3.0 10-^13^	gaagtgct	7.2 10^-9^	gaagtgct	4.6 10^-8^	gcgcatcc	6.8 10^-4^
tggcgctg*	5.8 10-^12^	tgaaactg*	4.0 10^-8^	ggcgctgg*	5.2 10^-8^	taggccgc	8.5 10^-4^
gcgctggt	7.2 10-^12^	atctggtg	4.9 10^-8^	cgatgaag	6.6 10^-8^	aagcttcg	1.1 10^-3^
cgctggtg	8.9 10^-12^	gcgctgga	8.0 10^-8^	tatctggt*	1.1 10^-7^	cgatgaag	1.1 10^-3^
cgcgctgg	1.0 10^-10^	cggtaaag	1.1 10^-7^	cggtaaag	1.4 10^-7^	cggataaa	1.2 10^-3^
gctggcga	1.3 10^-10^	ggttgatg*	1.4 10^-7^	ggttgatg*	2.0 10^-7^	ggggggac	1.4 10^-3^
tggcgcag	1.7 10^-10^	gtgctgga	1.6 10^-7^	gtgctgga	2.5 10^-7^	caggcgtt	1.6 10^-3^
ctggaaga	3.1 10^-10^	aattgtcg	2.1 10^-7^	tgggcttc	5.6 10^-7^	acgccttc	1.8 10^-3^

**Table 6:** Top: numbers of octamers significantly more exceptional in the loops when adjusting for a False Discovery Rate of 1% and associated thresholds for the p-value *p*_*B *_for different models. Bottom: idem for octamers significantly more exceptional in the backbone.

Model	M00	M0	M1	M6
Nb. of significant octamers	677	257	154	0
Threshold for *p*_*B*_	1.0 10^-4^	3.9 10^-5^	2.2 10^-5^	_

Nb. of significant octamers	159	23	14	0
Threshold for p′B MathType@MTEF@5@5@+=feaafiart1ev1aaatCvAUfKttLearuWrP9MDH5MBPbIqV92AaeXatLxBI9gBaebbnrfifHhDYfgasaacH8akY=wiFfYdH8Gipec8Eeeu0xXdbba9frFj0=OqFfea0dXdd9vqai=hGuQ8kuc9pgc9s8qqaq=dirpe0xb9q8qiLsFr0=vr0=vr0dc8meaabaqaciaacaGaaeqabaqabeGadaaakeaacuWGWbaCgaqbamaaBaaaleaacqWGcbGqaeqaaaaa@2F5A@	2.4 10^-5^	3.4 10^-6^	1.8 10^-6^	-

Symmetrically, to find the motifs with an exceptionality coefficient significantly higher in the backbone than in the loops, we have to test **H**_0 _versus H′1
 MathType@MTEF@5@5@+=feaafiart1ev1aaatCvAUfKttLearuWrP9MDH5MBPbIqV92AaeXatLxBI9gBaebbnrfifHhDYfgasaacH8akY=wiFfYdH8Gipec8Eeeu0xXdbba9frFj0=OqFfea0dXdd9vqai=hGuQ8kuc9pgc9s8qqaq=dirpe0xb9q8qiLsFr0=vr0=vr0dc8meaabaqaciaacaGaaeqabaqabeGadaaakeaaieqacuWFibasgaqbamaaBaaaleaacqaIXaqmaeqaaaaa@2EF3@ = {*k*_2 _> *k*_1_} using the *p*-value p′B
 MathType@MTEF@5@5@+=feaafiart1ev1aaatCvAUfKttLearuWrP9MDH5MBPbIqV92AaeXatLxBI9gBaebbnrfifHhDYfgasaacH8akY=wiFfYdH8Gipec8Eeeu0xXdbba9frFj0=OqFfea0dXdd9vqai=hGuQ8kuc9pgc9s8qqaq=dirpe0xb9q8qiLsFr0=vr0=vr0dc8meaabaqaciaacaGaaeqabaqabeGadaaakeaacuWGWbaCgaqbamaaBaaaleaacqWGcbGqaeqaaaaa@2F5A@ defined as p′B
 MathType@MTEF@5@5@+=feaafiart1ev1aaatCvAUfKttLearuWrP9MDH5MBPbIqV92AaeXatLxBI9gBaebbnrfifHhDYfgasaacH8akY=wiFfYdH8Gipec8Eeeu0xXdbba9frFj0=OqFfea0dXdd9vqai=hGuQ8kuc9pgc9s8qqaq=dirpe0xb9q8qiLsFr0=vr0=vr0dc8meaabaqaciaacaGaaeqabaqabeGadaaakeaacuWGWbaCgaqbamaaBaaaleaacqWGcbGqaeqaaaaa@2F5A@ = Pr{ℬ
 MathType@MTEF@5@5@+=feaafiart1ev1aaatCvAUfKttLearuWrP9MDH5MBPbIqV92AaeXatLxBI9gBaebbnrfifHhDYfgasaacH8akY=wiFfYdH8Gipec8Eeeu0xXdbba9frFj0=OqFfea0dXdd9vqai=hGuQ8kuc9pgc9s8qqaq=dirpe0xb9q8qiLsFr0=vr0=vr0dc8meaabaqaciaacaGaaeqabaqabeGadaaakeaat0uy0HwzTfgDPnwy1egaryqtHrhAL1wy0L2yHvdaiqaacqWFSeIqaaa@377D@ (*n*_+_, *π*_0_) ≤ *n*_1_}. The 10 most significant motifs for this test are given at the bottom of Table 5. When adjusting for a False Discovery Rate of 1%, only 14 octamers under model M1 are significantly more exceptional in the backbone than in the loops. These octamers have all a *p*-value p′B
 MathType@MTEF@5@5@+=feaafiart1ev1aaatCvAUfKttLearuWrP9MDH5MBPbIqV92AaeXatLxBI9gBaebbnrfifHhDYfgasaacH8akY=wiFfYdH8Gipec8Eeeu0xXdbba9frFj0=OqFfea0dXdd9vqai=hGuQ8kuc9pgc9s8qqaq=dirpe0xb9q8qiLsFr0=vr0=vr0dc8meaabaqaciaacaGaaeqabaqabeGadaaakeaacuWGWbaCgaqbamaaBaaaleaacqWGcbGqaeqaaaaa@2F5A@ smaller than 1.8 10^-6^. Note that under model M6, no octamer is significant after multiple testing adjustment.

According to the *p*_*B *_list, the motif cagcgctg has rank 1 115 among 65 536 under the M1 model. Note that the well known Chi motif (gctggtgg) which is the most overrepresented octamer in *E. coli *genome has a p′B
 MathType@MTEF@5@5@+=feaafiart1ev1aaatCvAUfKttLearuWrP9MDH5MBPbIqV92AaeXatLxBI9gBaebbnrfifHhDYfgasaacH8akY=wiFfYdH8Gipec8Eeeu0xXdbba9frFj0=OqFfea0dXdd9vqai=hGuQ8kuc9pgc9s8qqaq=dirpe0xb9q8qiLsFr0=vr0=vr0dc8meaabaqaciaacaGaaeqabaqabeGadaaakeaacuWGWbaCgaqbamaaBaaaleaacqWGcbGqaeqaaaaa@2F5A@ value of 5.1 10^-5 ^(rank 1 281) under the same model; It means that *k*_backbone _is significantly higher than *k*_loops _but due to multiple testing Chi is not among the significant octamers.

#### LRT versus binomial

We now compare the results provided by the two tests: binomial and LRT. Because the former is one-sided and the latter is two-sided, we use a signed version *LRT*^*s *^of the LRT statistic which is equal to *LRT *when *N*_1 _is greater than expected (*N*_1 _≥ *π*_0 _*N*_+_) and to – *LRT *otherwise (*N*_1 _<*π*_0 _*N*_+_). To make the graph more readable, we also transform the *p*-value *p*_*B *_into a Gaussian score *S*_*B *_∈ ℝ:

*S*_*B *_= *Φ*^-1 ^(1 - *p*_*B*_)

where Φ is the cumulative distribution function of the standard Gaussian distribution. High positive values of *S*_*B *_correspond to motifs with an exceptionality coefficient in sequence 1 significantly higher than in sequence 2, while high negative values of *S*_*B *_correspond to motifs having an exceptionality coefficient in sequence 1 significantly lower than in sequence 2.

**Figure 5:**
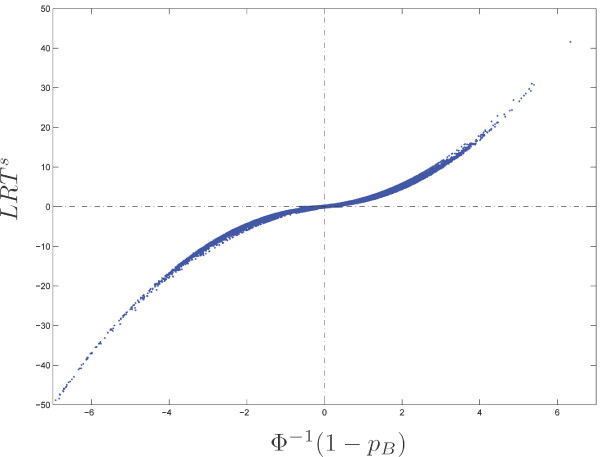
Signed LRT statistic *LRT*^*s *^(*y*-axis) versus transformed binomial *p*-value Φ^-1 ^(1 - *p*_*B*_) (*x*-axis) under model M1 for all octamers in the *E. coli *backbone/loops comparison.

We see in Figure 5 that the two statistics give very similar results for all the octamers in the backbone/loops comparison. Table 7 gives the Spearman and Kendall correlation coefficients between the two statistics for different models. Recall that Spearman's coefficient is the correlation between the ranks, while Kendall's one is the proportion of concordant pairs between the two rankings. This confirms that the LRT statistics is a reliable exceptionality comparison score, although the associated *p*-value is questionable for small counts.

**Table 7:** Spearman and Kendall correlation coefficients between *LRT*^*s *^and *S*_*B *_for different models.

Model	M00	M0	M1	M6
Spearman (%)	99.7	99.7	99.7	99.3
Kendall (%)	96.0	95.6	95.5	93.3

**Figure 6:**
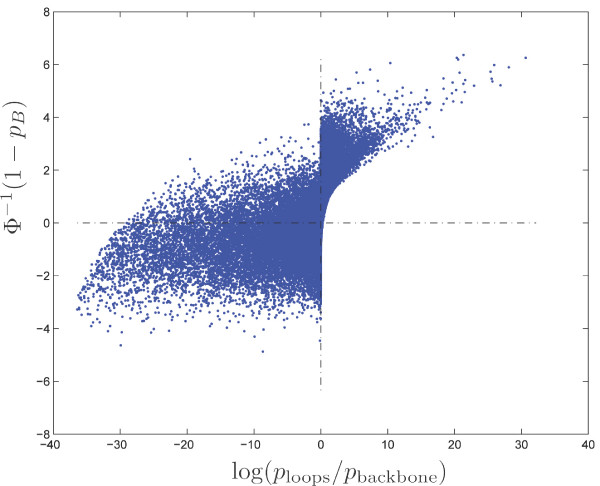
Transformed binomial *p*-value Φ^-1 ^(1 - *p*_*B*_) (*y*-axis) versus log ratio between the two *p*-values associated with the exceptionality of the motif in each sequence (*x*-axis) under model M1 for all octamers in the *E. coli *backbone/loops comparison.

Note that the naive comparison between the two *p*-values simply associated with the exceptionality of each motif in each sequence does not provide the same sets of significant octamers (see Figure 6). Such *p*-values have been calculated using the Poisson approximation of the number of clumps.

#### Test for overlaps

Very few motifs have significant differences in their clumps sizes. Table 8 presents the results for the 4 motifs having a *p*-value smaller than 10%. For all of them, no overlap is observed in the backbone (*C*_2 _= *N*_2 _means that all clumps are of size 1 while few are observed in the loops (*C*_1 _<*N*_1_). The probability *a *is the overlapping probability under model M00.

**Table 8:** Octamers with significant differences in terms of overlaps in the backbone/loops comparison.

Motif	Loops		backbone		*p*	a
	*C*_1_	*N*_1_	*C*_2_	*N*_2_	(%)	(%)
accactac	7	9	44	44	2.20	0.02
tattatta	38	41	69	69	4.83	1.56
tcggggtc	2	3	24	24	8.00	0.02
cgcgccgc	27	28	246	246	9.93	0.10

## Conclusion

We have proposed two complementary statistical tests to compare the exceptionalities of motif counts in two sequences. The binomial test is exact and particularly of interest for small counts (from a computational point of view). For large counts, we advise to use the likelihood ratio test which is asymptotic but strongly correlated with the exact binomial test. The LRT statistics is simple to calculate and can be directly interpreted as a contrast measure between the exceptionalities; its *p*-value can be derived from the chi-square distribution. Both tests will be implemented in the R'MES software already devoted to exceptional motifs [18].

The likelihood ratio test can be generalized to more than two sequences. Suppose we want to compare *I *sequences *S*_1_, *S*_2_,..., *S*_*I*_. In each of them, we assume that the count *N*_*i *_has a Poisson distribution with parameter *λ*_*i *_= *k*_*i *_ℓ_*i *_*μ*_*i *_and we want to test **H**_0 _= {*k*_1 _= *k*_2 _= ⋯ = *k*_*I*_} versus **H**_1 _= {At least one *k*_*i *_differs from the others}. The *LRT *statistics is

LRT=2∑iNiln⁡(NiN+∑jμjℓjμiℓi).
 MathType@MTEF@5@5@+=feaafiart1ev1aaatCvAUfKttLearuWrP9MDH5MBPbIqV92AaeXatLxBI9gBaebbnrfifHhDYfgasaacH8akY=wiFfYdH8Gipec8Eeeu0xXdbba9frFj0=OqFfea0dXdd9vqai=hGuQ8kuc9pgc9s8qqaq=dirpe0xb9q8qiLsFr0=vr0=vr0dc8meaabaqaciaacaGaaeqabaqabeGadaaakeaacqWGmbatcqWGsbGucqWGubavcqGH9aqpcqaIYaGmdaaeqbqaaiabd6eaonaaBaaaleaacqWGPbqAaeqaaaqaaiabdMgaPbqab0GaeyyeIuoakiGbcYgaSjabc6gaUnaabmaabaWaaSaaaeaacqWGobGtdaWgaaWcbaGaemyAaKgabeaaaOqaaiabd6eaonaaBaaaleaacqGHRaWkaeqaaaaakmaalaaabaWaaabeaeaaiiGacqWF8oqBdaWgaaWcbaGaemOAaOgabeaakiabloriSnaaBaaaleaacqWGQbGAaeqaaaqaaiabdQgaQbqab0GaeyyeIuoaaOqaaiab=X7aTnaaBaaaleaacqWGPbqAaeqaaOGaeS4eHW2aaSbaaSqaaiabdMgaPbqabaaaaaGccaGLOaGaayzkaaGaeiOla4caaa@51DF@

Under **H**_0_, *LRT *has an asymptotic chi-square distribution with (*I *- 1) degrees of freedom. The Chi-square test can be generalized as well.

Under the Poisson model, both tests can be easily used for degenerated motifs or more generally for sets of motifs. Let denote by W
 MathType@MTEF@5@5@+=feaafiart1ev1aaatCvAUfKttLearuWrP9MDH5MBPbIqV92AaeXatLxBI9gBaebbnrfifHhDYfgasaacH8akY=wiFfYdH8Gipec8Eeeu0xXdbba9frFj0=OqFfea0dXdd9vqai=hGuQ8kuc9pgc9s8qqaq=dirpe0xb9q8qiLsFr0=vr0=vr0dc8meaabaqaciaacaGaaeqabaqabeGadaaakeaat0uy0HwzTfgDPnwy1egaryqtHrhAL1wy0L2yHvdaiqaacqWFwe=vaaa@384C@ a set of motifs; The count *N*_*i *_(respectively the occurrence probability *μ*_*i*_) will be the sum of the counts (resp. occurrence probability) of **w **for all motifs **w **from W
 MathType@MTEF@5@5@+=feaafiart1ev1aaatCvAUfKttLearuWrP9MDH5MBPbIqV92AaeXatLxBI9gBaebbnrfifHhDYfgasaacH8akY=wiFfYdH8Gipec8Eeeu0xXdbba9frFj0=OqFfea0dXdd9vqai=hGuQ8kuc9pgc9s8qqaq=dirpe0xb9q8qiLsFr0=vr0=vr0dc8meaabaqaciaacaGaaeqabaqabeGadaaakeaat0uy0HwzTfgDPnwy1egaryqtHrhAL1wy0L2yHvdaiqaacqWFwe=vaaa@384C@. However, the generalization is much more involved for the compound Poisson model because of the possible overlaps between motifs from the set; In particular, the overlapping probability *a*_*i *_becomes a matrix [19].

We emphasize that these tests are valid only for independent sequences. They can not be used to detect skewed oligomers because the leading strand is not independent from the lagging strand [20]. This particular question requires the development of another rigorous statistical method; this is an ongoing work.

Finally, note that the exceptionality comparison of word counts in sequences is actually equivalent to the differential analysis of SAGE expression data [21]. Indeed, in the SAGE technology, the expression level of a given gene is measured by a number of associated tags and the problem is to compare the number of tags between two conditions. In such problem, no correction has to be done except for the total number of tags and our test statistics under model M00 are adapted.

## Methods

### Likelihood ratio test

The model presented in the Results Section can be rephrased as two Poisson processes with respective intensity *k*_*i *_*u*_*i *_(*i *= 1,2). To calculate the likelihood, we need to estimate the exceptionality coefficients *k*_1 _and *k*_2_. Under the alternative hypothesis, their respective maximum likelihood estimates (MLE) are k^
 MathType@MTEF@5@5@+=feaafiart1ev1aaatCvAUfKttLearuWrP9MDH5MBPbIqV92AaeXatLxBI9gBaebbnrfifHhDYfgasaacH8akY=wiFfYdH8Gipec8Eeeu0xXdbba9frFj0=OqFfea0dXdd9vqai=hGuQ8kuc9pgc9s8qqaq=dirpe0xb9q8qiLsFr0=vr0=vr0dc8meaabaqaciaacaGaaeqabaqabeGadaaakeaadaqiaaqaaiabdUgaRbGaayPadaaaaa@2ECD@_1 _= *N*_1_/(ℓ_1 _*μ*_1_) and k^
 MathType@MTEF@5@5@+=feaafiart1ev1aaatCvAUfKttLearuWrP9MDH5MBPbIqV92AaeXatLxBI9gBaebbnrfifHhDYfgasaacH8akY=wiFfYdH8Gipec8Eeeu0xXdbba9frFj0=OqFfea0dXdd9vqai=hGuQ8kuc9pgc9s8qqaq=dirpe0xb9q8qiLsFr0=vr0=vr0dc8meaabaqaciaacaGaaeqabaqabeGadaaakeaadaqiaaqaaiabdUgaRbGaayPadaaaaa@2ECD@_2 _= *N*_2_/(ℓ_2 _*μ*_2_). Assuming that the two sequences are independent, the log-likelihood of the two processes is

ℒ1=∑i=12[Niln⁡(k^iμi)−k^iμiℓi]=∑i=12[Niln⁡(Ni/ℓi)−Ni].
 MathType@MTEF@5@5@+=feaafiart1ev1aaatCvAUfKttLearuWrP9MDH5MBPbIqV92AaeXatLxBI9gBaebbnrfifHhDYfgasaacH8akY=wiFfYdH8Gipec8Eeeu0xXdbba9frFj0=OqFfea0dXdd9vqai=hGuQ8kuc9pgc9s8qqaq=dirpe0xb9q8qiLsFr0=vr0=vr0dc8meaabaqaciaacaGaaeqabaqabeGadaaakeaafaqadeGabaaabaWenfgDOvwBHrxAJfwnHbqeg0uy0HwzTfgDPnwy1aaceaGae8NeHW0aaSbaaSqaaiabigdaXaqabaGccqGH9aqpdaaeWbqaamaadmaabaGaemOta40aaSbaaSqaaiabdMgaPbqabaGccyGGSbaBcqGGUbGBcqGGOaakdaqiaaqaaiabdUgaRbGaayPadaWaaSbaaSqaaiabdMgaPbqabaacciGccqGF8oqBdaWgaaWcbaGaemyAaKgabeaakiabcMcaPiabgkHiTmaaHaaabaGaem4AaSgacaGLcmaadaWgaaWcbaGaemyAaKgabeaakiab+X7aTnaaBaaaleaacqWGPbqAaeqaaOGaeS4eHW2aaSbaaSqaaiabdMgaPbqabaaakiaawUfacaGLDbaaaSqaaiabdMgaPjabg2da9iabigdaXaqaaiabikdaYaqdcqGHris5aaGcbaGaeyypa0ZaaabCaeaacqGGBbWwcqWGobGtdaWgaaWcbaGaemyAaKgabeaakiGbcYgaSjabc6gaUjabcIcaOiabd6eaonaaBaaaleaacqWGPbqAaeqaaOGaei4la8IaeS4eHW2aaSbaaSqaaiabdMgaPbqabaGccqGGPaqkcqGHsislcqWGobGtdaWgaaWcbaGaemyAaKgabeaakiabc2faDjabc6caUaWcbaGaemyAaKMaeyypa0JaeGymaedabaGaeGOmaidaniabggHiLdaaaaaa@7716@

Under the null hypothesis, the common MLE of *k*_1 _and *k*_2 _is k^
 MathType@MTEF@5@5@+=feaafiart1ev1aaatCvAUfKttLearuWrP9MDH5MBPbIqV92AaeXatLxBI9gBaebbnrfifHhDYfgasaacH8akY=wiFfYdH8Gipec8Eeeu0xXdbba9frFj0=OqFfea0dXdd9vqai=hGuQ8kuc9pgc9s8qqaq=dirpe0xb9q8qiLsFr0=vr0=vr0dc8meaabaqaciaacaGaaeqabaqabeGadaaakeaadaqiaaqaaiabdUgaRbGaayPadaaaaa@2ECD@ = (*N*_1 _+ *N*_2_)/(ℓ_1 _*μ*_1 _+ ℓ_2 _*μ*_2_) and the log-likelihood is

ℒ0=∑i=12[Niln⁡(k^μi)−k^μiℓi]=∑i=12[Niln⁡(k^μi)−Ni].
 MathType@MTEF@5@5@+=feaafiart1ev1aaatCvAUfKttLearuWrP9MDH5MBPbIqV92AaeXatLxBI9gBaebbnrfifHhDYfgasaacH8akY=wiFfYdH8Gipec8Eeeu0xXdbba9frFj0=OqFfea0dXdd9vqai=hGuQ8kuc9pgc9s8qqaq=dirpe0xb9q8qiLsFr0=vr0=vr0dc8meaabaqaciaacaGaaeqabaqabeGadaaakeaat0uy0HwzTfgDPnwy1egaryqtHrhAL1wy0L2yHvdaiqaacqWFsectdaWgaaWcbaGaeGimaadabeaakiabg2da9maaqahabaWaamWaaeaacqWGobGtdaWgaaWcbaGaemyAaKgabeaakiGbcYgaSjabc6gaUjabcIcaOmaaHaaabaGaem4AaSgacaGLcmaaiiGacqGF8oqBdaWgaaWcbaGaemyAaKgabeaakiabcMcaPiabgkHiTmaaHaaabaGaem4AaSgacaGLcmaacqGF8oqBdaWgaaWcbaGaemyAaKgabeaakiabloriSnaaBaaaleaacqWGPbqAaeqaaaGccaGLBbGaayzxaaaaleaacqWGPbqAcqGH9aqpcqaIXaqmaeaacqaIYaGma0GaeyyeIuoakiabg2da9maaqahabaWaamWaaeaacqWGobGtdaWgaaWcbaGaemyAaKgabeaakiGbcYgaSjabc6gaUjabcIcaOmaaHaaabaGaem4AaSgacaGLcmaacqGF8oqBdaWgaaWcbaGaemyAaKgabeaakiabcMcaPiabgkHiTiabd6eaonaaBaaaleaacqWGPbqAaeqaaaGccaGLBbGaayzxaaaaleaacqWGPbqAcqGH9aqpcqaIXaqmaeaacqaIYaGma0GaeyyeIuoakiabc6caUaaa@7263@

The LRT is defined as twice the difference between ℒ
 MathType@MTEF@5@5@+=feaafiart1ev1aaatCvAUfKttLearuWrP9MDH5MBPbIqV92AaeXatLxBI9gBaebbnrfifHhDYfgasaacH8akY=wiFfYdH8Gipec8Eeeu0xXdbba9frFj0=OqFfea0dXdd9vqai=hGuQ8kuc9pgc9s8qqaq=dirpe0xb9q8qiLsFr0=vr0=vr0dc8meaabaqaciaacaGaaeqabaqabeGadaaakeaat0uy0HwzTfgDPnwy1egaryqtHrhAL1wy0L2yHvdaiqaacqWFsectaaa@376D@_1 _and ℒ
 MathType@MTEF@5@5@+=feaafiart1ev1aaatCvAUfKttLearuWrP9MDH5MBPbIqV92AaeXatLxBI9gBaebbnrfifHhDYfgasaacH8akY=wiFfYdH8Gipec8Eeeu0xXdbba9frFj0=OqFfea0dXdd9vqai=hGuQ8kuc9pgc9s8qqaq=dirpe0xb9q8qiLsFr0=vr0=vr0dc8meaabaqaciaacaGaaeqabaqabeGadaaakeaat0uy0HwzTfgDPnwy1egaryqtHrhAL1wy0L2yHvdaiqaacqWFsectaaa@376D@_0_: *LRT *= 2(ℒ
 MathType@MTEF@5@5@+=feaafiart1ev1aaatCvAUfKttLearuWrP9MDH5MBPbIqV92AaeXatLxBI9gBaebbnrfifHhDYfgasaacH8akY=wiFfYdH8Gipec8Eeeu0xXdbba9frFj0=OqFfea0dXdd9vqai=hGuQ8kuc9pgc9s8qqaq=dirpe0xb9q8qiLsFr0=vr0=vr0dc8meaabaqaciaacaGaaeqabaqabeGadaaakeaat0uy0HwzTfgDPnwy1egaryqtHrhAL1wy0L2yHvdaiqaacqWFsectaaa@376D@_1 _- ℒ
 MathType@MTEF@5@5@+=feaafiart1ev1aaatCvAUfKttLearuWrP9MDH5MBPbIqV92AaeXatLxBI9gBaebbnrfifHhDYfgasaacH8akY=wiFfYdH8Gipec8Eeeu0xXdbba9frFj0=OqFfea0dXdd9vqai=hGuQ8kuc9pgc9s8qqaq=dirpe0xb9q8qiLsFr0=vr0=vr0dc8meaabaqaciaacaGaaeqabaqabeGadaaakeaat0uy0HwzTfgDPnwy1egaryqtHrhAL1wy0L2yHvdaiqaacqWFsectaaa@376D@_0_). The result follows after standard algebraic manipulations.

## Appendix

### Exact hyper-geometric test

#### Conditional distribution of the number of clumps

The conditional distribution of *C*_*i *_- 1 given in (2) can be modified as

*N*_*i *_- *C*_*i *_~ ℬ
 MathType@MTEF@5@5@+=feaafiart1ev1aaatCvAUfKttLearuWrP9MDH5MBPbIqV92AaeXatLxBI9gBaebbnrfifHhDYfgasaacH8akY=wiFfYdH8Gipec8Eeeu0xXdbba9frFj0=OqFfea0dXdd9vqai=hGuQ8kuc9pgc9s8qqaq=dirpe0xb9q8qiLsFr0=vr0=vr0dc8meaabaqaciaacaGaaeqabaqabeGadaaakeaat0uy0HwzTfgDPnwy1egaryqtHrhAL1wy0L2yHvdaiqaacqWFSeIqaaa@377D@ (*N*_*i *_- 1, *b*_*i*_)

where *b*_*i *_= *h*_*i*_*a*_*i *_is the true overlapping probability. This version is preferable, since the exceptionality coefficient *h*_*i *_directly appears here as a multiplicative constant. The conditional distribution of the difference *N*_*i *_- *C*_*i *_given the clump counts *C*_1 _and *C*_2 _and the total count *N*_+ _is a generalized negative hyper-geometric distribution (see [12] p. 264 for the classical version and p. 270 for the generalization):

Pr⁡{N1=n1|C1,C2,N+}=A−1(n1−1C1−1)(N+−n1−1C2−1)(N+−2C+−2)(b1b2)n1−C1
 MathType@MTEF@5@5@+=feaafiart1ev1aaatCvAUfKttLearuWrP9MDH5MBPbIqV92AaeXatLxBI9gBaebbnrfifHhDYfgasaacH8akY=wiFfYdH8Gipec8Eeeu0xXdbba9frFj0=OqFfea0dXdd9vqai=hGuQ8kuc9pgc9s8qqaq=dirpe0xb9q8qiLsFr0=vr0=vr0dc8meaabaqaciaacaGaaeqabaqabeGadaaakeaafaqadeGabaaabaGagiiuaaLaeiOCaiNaei4EaSNaemOta40aaSbaaSqaaiabigdaXaqabaGccqGH9aqpcqWGUbGBdaWgaaWcbaGaeGymaedabeaakiabcYha8jabdoeadnaaBaaaleaacqaIXaqmaeqaaOGaeiilaWIaem4qam0aaSbaaSqaaiabikdaYaqabaGccqGGSaalcqWGobGtdaWgaaWcbaGaey4kaScabeaakiabc2ha9jabg2da9aqaaiabdgeabnaaCaaaleqabaGaeyOeI0IaeGymaedaaOWaaSaaaeaadaqadaqaauaabeqaceaaaeaacqWGUbGBdaWgaaWcbaGaeGymaedabeaakiabgkHiTiabigdaXaqaaiabdoeadnaaBaaaleaacqaIXaqmaeqaaOGaeyOeI0IaeGymaedaaaGaayjkaiaawMcaamaabmaabaqbaeqabiqaaaqaaiabd6eaonaaBaaaleaacqGHRaWkaeqaaOGaeyOeI0IaemOBa42aaSbaaSqaaiabigdaXaqabaGccqGHsislcqaIXaqmaeaacqWGdbWqdaWgaaWcbaGaeGOmaidabeaakiabgkHiTiabigdaXaaaaiaawIcacaGLPaaaaeaadaqadaqaauaabeqaceaaaeaacqWGobGtdaWgaaWcbaGaey4kaScabeaakiabgkHiTiabikdaYaqaaiabdoeadnaaBaaaleaacqGHRaWkaeqaaOGaeyOeI0IaeGOmaidaaaGaayjkaiaawMcaaaaadaqadaqaamaalaaabaGaemOyai2aaSbaaSqaaiabigdaXaqabaaakeaacqWGIbGydaWgaaWcbaGaeGOmaidabeaaaaaakiaawIcacaGLPaaadaahaaWcbeqaaiabd6gaUnaaBaaameaacqaIXaqmaeqaaSGaeyOeI0Iaem4qam0aaSbaaWqaaiabigdaXaqabaaaaaaaaaa@73A3@

where *A *is the constant such that the sum over all *n*_1 _between *C*_1 _and *N*_+ _is equal to one.

#### Test

Under **H**_0 _= {*h*_1 _= *h*_2_}, the term *b*_1_/*b*_2 _can be replaced by *a*_1_/*a*_2_. The overlapping probability *b*_1 _is significantly greater than *b*_2 _if *N*_1 _is significantly large, *i.e*. if Pr{*N*_1 _≥ *n*_1_|*C*_1_, *C*_2_, *N*_+_} is small. The power of this test can also be studied: under **H**_0_, *b*_1_/*b*_2 _equals *a*_1_/*a*_2_, while under the alternative hypothesis, it is equal to (*h*_1_/*h*_2_) (*a*_1_/*a*_2_). The power of the test is then a function of *h*_1_/*h*_2_.

## Authors' contributions

SR and SS developed the statistical methodology, analyzed the examples and wrote the paper. VV studied the usage conditions. All authors read and approved the final manuscript.
